# Progress of Veterinary Science

**Published:** 1882-10

**Authors:** 


### PROGRESS OF VETERINARY SCIENCE.

An English slaughterman makes the statement that but few calves have perfectly healthy livers, "not one in ten," most of them containing larger or smaller collections of putrid "matter."

Camel's Lung with Filariæ Sanguinis.—Mr. Eve exhibited before the London Pathological Society, April 18th, a specimen which showed adult filariæ sanguinis in the lung of a camel. The animal had suffered from a wasting disease for a year, and every drop of blood was found to contain ten or twelve embryo filariæ. The camel was killed by decapitation, and on examination adult filariæ were found in tangled masses within the aorta and the pulmonary artery and branches. Dr. Lewis, to whom the specimens had been sent, said that though the embryo was like that of the filaria sanguinis hominis, the adult was much larger, and evidently of a different species.

A Blood Diet for the Herbivora.—The fact that all animals, even the herbivora, are at first nourished on a diet of milk, which must be considered as purely animal, led M. Regnard to suggest that an animal diet might be employed to advantage at a subsequent epoch in their history. The idea is not new, but attempts to verify it by, for instance, feeding horses on meat, raw or cooked, invariably failed in consequence of the disgust which this diet occasioned. It occurred, however, to M. Regnard that an animal substance might be utilized—certainly highly nutritious, of which tons are wasted weekly—viz., blood. The problem was how to present it to the animal in such a form as not to occasion disgust. The blood was heated to 100°C., and the coagulum thus obtained was pressed and rapidly dried in a stove, and then powdered in a coffee mill. It was found to keep well, and to be destitute of odor and of taste, and was given mixed with other food in doses varying from ten to eighty grammes daily. The experiments were made on lambs which had been abandoned by their mothers. Three lambs were kept on the ordinary diet of beetroot, hay, etc., and to three others the powdered blood was given. The first steadily lost flesh, while the latter increased to three times the original weight, and connoisseurs declared that they had never seen such fine lambs of the same age. The animals surpassed their fellows which had been suckled by their dams both in weight and size, and their coat of wool was doubled in thickness. Experiments of the same kind are now in progress with calves, and promise to be as successful as the others. It will certainly be a matter of high importance if the vast amount of nutriment now almost entirely wasted can be thus utilized. The saving of milk in rearing calves, for instance, would alone be a most valuable item. It appears that this system of alimentation is applicable also to man. In the case of a rickety child of eighteen months the results are said to have been most encouraging.

THE SALMON DISEASE AND ITS LESSONS.—Professor Huxley has published some observations on the epidemic known as the Salmon Disease in the proceedings of the Royal Society. The disease, as is well-known, is produced by the growth of a parasitic fungus, and Professor Huxley looks upon it as a disease of the same order as ringworm in the human subject, as the muscardine of silkworms, and the potato-disease. This fungus, which belongs to the order *Saprolegnia*, finds a suitable nidus in the skin of that part of the body which is devoid of scales, and generally first attacks the top and sides of the head; thence it may extend widely over the scaly surface also, and deeply into the true skin, causing extensive ulceration and sloughing, so that "one vast open sore may cover the top of the head from the snout to the nape, and may extend over the gill-covers." Several points of general interest have come out in the course of the inquiry; one of these is, that the fungus does not attack the viscera, so that the flesh of the diseased fish is probably not injurious in any way; and it has been said, by those who have made the experiment, that the palate can detect no difference between it and the flesh of a healthy fish. This applies probably only to the early stage; for when death—which is produced by exhaustion—is approaching, the flesh no doubt deteriorates in quality. Another interesting point is the manner in which the sloughing of the true skin is produced. The fungus at first attacks the cuticle, but, after it has taken root there, it sends processes (hyphæ) downwards into the true derma; these processes branch laterally in every direction, and gradually extend deeper and deeper. The tracks of these hyphæ are not accompanied by any obvious inflammation; but they are so closely set that they mechanically interfere wlth the nutrition of the part, and so lead to sloughing. The third point to which we wish to draw attention is, that the fungus is essentially a saprophyte—*i.e.*, it ordinarily finds its nidus in dead animals or vegetable tissues, and is only occasionally a parasite upon living organisms. Every stream in the kingdom probably contains indefinite quantities of this and allied fungi, whieh grows readily on the bodies of dead flies and other insects. Professor Huxley thus arrives at a conclusion with regard to this disease analagous to that to which the student of human pathology is often brought in the case of many infectious diseases—namely, that though the parasitic organism may be the determining cause of the train of symptoms which come under observation, there are other, and as yet unknown, circumstances, extrinsic or intrinsic to the infected animal, in the absence of which the parasite cannot develop.

